# Usefulness of MRI in the follow-up of ERA-related sacroiliitis, and effectiveness of treatment with sulphasalazine

**DOI:** 10.1186/1546-0096-9-S1-P271

**Published:** 2011-09-14

**Authors:** Ilaria Pagnini, Gabriele Simonini, Teresa Giani, Sara Savelli, Claudio Fonda, Rolando Cimaz

**Affiliations:** 1 Department of Pediatric, University of Florence, Italy

## Background

Dynamic-MRI is the gold-standard to evaluate the early involvement of the sacroiliac joints in patients with enthesitis-related arthritis (ERA).

## Aim

In our study, this method was also used to define the evolution over time of sacroiliitis (SI) and to evaluate the response to therapy.

## Methods

A retrospective chart review for all patients with SI followed in our Unit since 2006 was performed. In all these patients the clinical, laboratory and instrumental follow-up was evaluated using dynamic contrast-enhanced MRI with fat suppression technique (STIR), in order to assess the radiological outcome and response to therapy.

## Results

47 patients (29 M, 18F), complained of inflammatory back pain, after a mean interval of 9 months (range 0 – 4 yrs11 m) from disease onset. In all symptomatic pts dynamic MRI of the sacroiliac joints was performed, and was positive in 28/47 (17 M, 11 F). We identified 25 pts with grade I SI (5 unilateral and 20 bilateral), 1 pt with grade II bilateral SI, and 2 pts with grade 2/I SI (right/left). In 16 patients (57%) treatment with sulphasalazine (SSZ) was started, in 11 (39%) with NSAIDs only, and in one with Adalimumab.

After 1 year, 12 pts remained symptomatic and MRI was repeated, and 6 still had radiological signs of SI, including 5 with bilateral SI grade I and one with grade II/I SI. They were on treatment with NSAIDs (n=2) and SSZ (n=4); the 6 pts in which MRI was negative were also treated with NSAIDs (n=2) and SSZ (n=4). Three of them still had a positive MRI after 2 years (all on SSZ).

## Conclusions

Dynamic contrast-enhanced MRI is useful not only to identify early involvement of sacroiliitis in patients with symptomatic ERA, but also to evaluate the course of the disease and response to therapy. Although SSZ is a commonly used drug for the treatment of SI, it was effective only in few of our cases.

